# Motivational techniques to improve self-care in hemophilia: the need to support autonomy in children

**DOI:** 10.1186/s12887-016-0542-9

**Published:** 2016-01-11

**Authors:** Sarah Bérubé, Florine Mouillard, Claudine Amesse, Serge Sultan

**Affiliations:** CHU Sainte-Justine, Université de Montreal, 3175 Chemin de la Côte-Sainte-Catherine, Montreal, QC H3T 1C5 Canada

**Keywords:** Hemophilia, Motivation, Adherence, Self-care, Intervention, Psychosocial, Self-determination

## Abstract

**Background:**

In pediatric hemophilia, caregivers are facing unique challenges to adherence and self-care in children and adolescents with hemophilia. Hemophilia treatment requires adequate prophylaxis and on-demand treatment, as well as a clear behavioral strategy to limit risk-taking in terms of physical exercise and diet. Medication adherence rates of hemophilia patients have been reported to decrease during late childhood and adolescence. In the developing child, moving safely from parent-care to self-care is one of the greatest challenges of integrative care within this domain. There is a clear need for initiatives designed to increase an individual’s motivation for treatment and self-care activities.

**Discussion:**

Among motivational approaches, the self-determination perspective offers a useful framework to explain how the transition to self-care can be facilitated. We discuss how motivation regarding hemophilia treatment may be increased through parental autonomy support and we offer examples of applied communication techniques to facilitate autonomy-supportive caregiving. Although it has not yet been tested in the context of hemophilia, these communication techniques could potentially help caregivers promote adherence and self-care in children.

**Summary:**

Confronted by unique challenges to adherence and self-care, caregivers of children with hemophilia should move from an exclusive focus on illness-management education to an integrative strategy, including motivation-enhancing communication. The self-determination perspective provides important proximal objectives (e.g. autonomy support) to maintain optimal adherence in adolescents as they move from parent-care to self-care. Future research initiatives should address the practice of these communication techniques and evaluate them in the context of hemophilia.

## Background

Hemophilia is a rare genetic disorder that can lead to bleeding episodes in the joints and muscles, which can result in permanent damages. Current treatment requires adequate routine administration of the missing coagulation factor (prophylaxis) or the administration of infusions when bleeding symptoms occurs (episodic treatment). Without adequate adherence, people with hemophilia tend to experience more frequent and severe pain and bleeding episodes as well as a lower quality of life [1, 2]. Research has consistently found that adherence to treatment tends to decrease during late childhood and adolescence in patients with other chronic health conditions [3]. The same tendency was observed in hemophilia where adherence to prophylaxis has been shown to decrease drastically during early adolescence, when self-administrating usually starts. In a recent survey of healthcare providers in 147 treatment centers around the world, 90 % of children with hemophilia aged 0–12 years had “high” or “very high” adherence to prophylaxis compared with only 54 % for those aged 13–18 years [4]. Reported barriers to adherence recommendations included such reasons as “lack of time” or “forgetfulness” [4–8]. Moreover, 25 % of young patients who were not “early treaters” when bleeding occurred, reported that they did not have the clotting agent in their possession at the time and 29 % stated that they did not recognize the symptoms of the episode [6]. These challenges could be partly addressed by offering patients and their family education in illness management skills. However, it has been shown that information interventions alone only have a negligible effect in pediatrics [9]. Clinicians also often recognize that these reported barriers are linked to motivational factors. Hemophilia patients may have only experienced a few bleeding episodes in their childhood due to adequate adherence to prophylaxis, as they were being treated by their parents. As adolescents, they may not realize the importance of preventive measures. This could lead to reduced adherence to prophylaxis recommendations or stopping their infusions [10]. Adolescents are also often described as being more present-oriented. Thus they may not be willing or capable of considering long-term consequences and may even ignore their disease because of their desire to be perceived as “normal” [3]. Adolescents may also be less inclined to listen to their parents and might want to test their limits [3]. Teenage rebellion, or the desire to free oneself from imposed constraints, has been described by nurses as a key factor in hemophilia non-adherence [4]. Motivational factors appear central to adherence and self-care behaviors so that patients do not see their treatment plan as being imposed on them by their caregivers.

Although fewer studies are available on self-care behaviors than on adherence to medical treatment, it is probable that the same motivational factors influence adolescents’ choices regarding physical activity (PA) and weight control. It has been shown that fostering motivation in patients can lead to better adherence to the recommended exercise [11]. The World Federation of Haemophilia guidelines recommend appropriate PA to help promote fitness, neuromuscular development, coordination and healthy body weight in order to prevent joint damage [12]. Non-contact sports such as swimming will be encouraged, while high contact/collision or high speed sports such as football or hockey will not be recommended for these patients [12]. Surprisingly and contrasting with the aforementioned guidelines, a national survey conducted in 2006 found that 60 % of 459 young patients across the US managed their hemophilia by simply avoiding physical activity altogether [6]. On the other hand, a higher proportion than expected had engaged in high-impact activities in the last year [13]. In a 2007 survey, 74 young patients with hemophilia in Germany were asked to indicate their primary motivations for participating in sports activities. “Having fun” or, “social aspects” were always chosen as the top two reasons for their participation, with “health benefits” consistently coming in third place [14].

Adherence issues have been observed in hemophilia as in many other chronic conditions, despite important advances made in education management training [4, 15–17]. This observation underlines the role of other important factors, such as motivation, when confronting adherence issues. As evidenced in more prevalent conditions such as diabetes, intervening on motivation and autonomy aspects may be highly effective in increasing adherence and self-care in pediatrics [18, 19]. Parents also need to receive sufficient support in order to help their child accept and adapt to their illness and its management. In the present article, we explain how parents and caregivers can implement motivational techniques to help their child or adolescent gradually and safely move towards greater independence. We offer practical communication strategies, which are likely to change motivation and thus increase adherence to the treatment regimen.

## Discussion

### Motivation for self-care

Various reasons, attitudes and beliefs may lead young patients to avoid general treatment recommendations, especially when they start self-infusions and making their own choices. Therefore, children and adolescents must be guided in developing and maintaining motivation for their treatment and health behaviors. This motivation should persist while parental supervision progressively decreases and the child start self-infusing, which usually occurs before the age of 15 [20]. Parents may understand the importance of following the recommendations and may become engaged in the management of their child’s illness in the very early stages, but this motivation also needs to be transferred to the child as he/she gains more autonomy. It can be difficult for parents to find a balance between prohibiting behaviours and encouraging their child’s autonomy and freedom of choice [21]. Overprotection as well can have detrimental psychosocial effects on children [22–25]. However, overindulgence is also associated with negative psychological effects, especially in the context of hemophilia where children need to avoid potentially dangerous behaviors [26]. Few programs formally help parents deal with everyday problems related to hemophilia management [27]. Typically, little information is usually given to parents to help them adequately address illness-related issues with their children, such as how to decrease the burden of injections or how to discuss risky sports involvements. If not addressed properly in early childhood, these issues may be perceived by children as restrictions to their autonomy, which could result in a lack of motivation to pursue appropriate behaviors when they are older.

### Self-determination in children

One approach psychologists have used to understand and promote motivation is called Self-Determination Theory (SDT) [28]. According to this approach, it is possible to set limits without precluding children from becoming inherently motivated for their behavior and thus motivated to participate in activities that are not necessarily pleasant, such as infusions [29]. According to this theory, people have a natural tendency to internalize socially transmitted values, but this internalization process can be supported or hindered by their environment [30]. This process may be viewed on a continuum ranging from being motivated by external factors (external regulation) to being motivated by internal factors (integrated regulation) [31]. A child would be motivated by external factors if he/she engages in the recommended activities in order to obtain parental approval, rewards or to avoid guilt [32]. This form of motivation has sometimes been associated with short-term benefits, but is often accompanied with anxiety and dissatisfaction and does not typically persist over time [33]. From a self-care perspective, it is desirable that young patients with hemophilia develop a more integrated form of regulation for their behaviors (e.g., engaging in physical activity because physical activity is important for one’s health and is in line with one’s core values). This type of motivation is more likely to guide adolescent into making healthy choices in the absence of parental supervision. SDT is empirically-based and has validated instruments to assess each of its constructs, which facilitates the development and evaluation of interventions (https://www.selfdeterminationtheory.org). Compared to other frameworks, such as motivational interviewing or empowerment, techniques from SDT can be incorporated into family dynamics and healthcare practices as a way to communicate to children about their illness and its management, and thus are specifically applicable to the developing child. It is also possible to use these communication tools with children of all ages. The sets of communication techniques derived from SDT aim at fulfilling an individual’s innate psychological needs for autonomy, competence and relatedness, which in turn can facilitate and foster a more integrated form of regulation [31]. Among these needs, autonomy would be essential in achieving a more integrated form of regulation and for this reason, many interventions are referred to as “autonomy-supportive” even though they also respond to needs for competence and relatedness [31]. SDT has been applied to chronic conditions requiring complex treatment regimens and was found to be effective for many outcomes, including adherence and physical activity [28]. A meta-analysis supported the development and implementation of interventions using this approach in patients of different ages [33]. It concluded that autonomy-support from healthcare providers predicts patients’ autonomy and satisfaction of needs, which in turn are important predictors of self-care and health outcomes [33]. A study has found that autonomy-support provided by parents can promote PA in children (e.g. pedometer-determined PA level) [34]. Besides the favorable outcomes for health-related behaviors, SDT is known to have both short and long-term impacts in a variety of areas, such as academic performance, psychological well-being and social adjustment [35–38]. On the contrary, a controlling family environment can undermine the development of an integrated form of regulation. Such control refers to the act of pressuring the child to think, feel or act differently using various techniques such as inducing guilt, withdrawing love or invalidating feelings [36]. Several reasons can lead parents to adopt such attitudes including anxiety and concerns about their child’s health status [39].

### Strategies to increase motivation in children with hemophilia

Autonomy-support should not be confused with permissiveness or neglect. Autonomy supportive environments typically include a clear structure for children in order to give them opportunities for self-achievement, all the while preventing them from engaging in risky behaviors [40]. Existing interventions have shown that professionals and parents can be trained to use an autonomy-supportive communication approach where the child is helped in meeting his or her innate psychological needs [41, 42]. This may be achieved by encouraging personal choices, independent problem-solving, taking initiative, and by participation in shared decision-making. In such environments, the child’s point of view is acknowledged as well as his/her ideas and feelings [28, 40, 43, 44]. However, this can represent a challenge with a child with hemophilia as the need to limit health risks strongly encourages caregivers to be more controlling than autonomy-supportive, thus emphasizing obedience or compliance. Unfortunately, with this latter type of approach, the child will generally be less adherent to his treatment or recommended self-care behaviors.

### Examples of motivation-focused communication

In order to illustrate how these motivational principles may be applied in practice, we have gathered examples of autonomy-supportive communications that could be used with children with hemophilia (Table 1). We illustrate four communication principles based on SDT as applied to real-life situations, which could be potentially encountered by these parents. The fear that children may hurt themselves may lead distressed parents to become overly controlling and protective, hence potentially removing all opportunities for the child to find his/her own solutions to daily challenges. Motivation research suggests that these children do not only need education in order to adequately limit their risks, but they also need to develop the feeling that they are responsible for their own behaviors [45]. Hence the structure given to children should be most effective if they can experience a sense of volition and choice. Importantly, research on self-determination suggests that any trained professional can communicate this way, and transfer these communication strategies to parents, either as part of routine clinical appointments or during more systematic interventions [46, 47]. Long-term management of haemophilia could greatly benefit from the development and the evaluation of such an approach.

**Table 1:** Application of autonomy support concepts to parenting a child with hemophilia

Topic 1: Acknowledging and being sensitive to his/her perspective, feelings and ideas Example: *The child comes back from school crying because his teacher said he/she could not play soccer during recess time.*			
Autonomy supportive		Non autonomy supportive	
1. Name the child’s feeling. Acknowledge that it can be difficult.	*“You must have felt really angry when the teacher said you could not play soccer.”*	Being judgmental about the child’s feelings and ideas. Rationalizing or minimizing emotions.	*“Stop acting like a baby, you know why, you know the teacher did that for your own good, you should be grateful.”*
2. Show that you are listening and let the child find his/her own solution	*“I see… Hmm Hmm…”*	Interrupting Criticizing	*“We have told you many times that you can’t play, you should not even start to play.”*
3. Encourage the child to propose ideas and write them down, even the ones that are not suitable with his/her health condition.	*“Let’s make a list of all the sports that you would like to play.”*	Making judgments about what he or she values as good or important	*“Health should be your priority.”*
4. Take into consideration his/her opinion about the suitability of the behaviour.	*“Let’s see which ones are possible, or not, for you to play and why.”*	Imposing your opinion.	*“I enrolled you in swimming classes.”*
5. Ask questions to find out what the child likes in this specific unsuitable sport? Help him/her to be specific when they do not like something.	*“What do you like in this sport? Is it being with your friends?”* *“Why don’t you like swimming?”*	Trying to convince.	“*We are lucky that we have a pool right next to our house, many kids would be happy to have that*.”
6. Help the child to find alternatives that meet these interests.	*“Let’s see what could make these activities safe for you. Do you have any ideas?”* “*Which other sport would make you go fast like when people play hockey?”*	Impose solutions	*“Next time, you should explain to your friend that you can’t play.”*
Topic 2: Providing choices, minimizing control and involving the child as much as possible Example: *The child does not want to receive his injection in the morning.*			
Autonomy supportive		Non autonomy supportive	
1. As much as possible, give the child choices related to the management of hemophilia.	*“Would you prefer to watch TV during your injection or read a book ?”*	Imposing decisions, applying pressure or arguing	*“The doctor said you have to receive your injection in the morning, if you do not I am going to tell him and he won’t be happy.”*
2. Engage the child as much as possible in his treatment	*“Do you want to disinfect your skin while I prepare your injection?”*	Being inconsistent or too permissive	“*It’s ok I give up, we will do your injection tomorrow.”*
Topic 3: Providing structure and explaining the rationale when choices are not possible Example: The child hurt himself/herself playing outside and did not tell anyone, which caused a bleeding episode.			
Autonomy supportive		Non autonomy supportive	
1. Explain in a language adapted to the child’s level of comprehension as to why the preventive behaviours are important.	“*You have to call mommy so we can inject the little soldiers in your blood that will fix the boo boo.”*	Giving too much information at once, accentuating long-term consequences or scaring the child.	“ *If you don’t receive your injection, you might not be able to walk when you are my age.”*
2. Set important limits for the child and stay consistent.	*“You always have to call me when you feel in pain and I will come and take care of your infusion.”*	Setting excessive rules.	*“You have to call me before engaging in any physical activity.”*
3. Encourage questions. Encourage the child’s ideas and for them to look for answers.	*“Do you know why your knee is getting bigger? Why don’t we look up on the internet to find out or we can call the nurse tomorrow?”*	Avoiding discussion	“*We have talked about it many times, you know what you have to do.”*
Topic 4: Showing compassion for the child and providing non-judgmental feedback			
Autonomy supportive		Non autonomy supportive	
1. Provide feedback that is not judgmental.	*“It’s a good thing that you called me, even though you felt ashamed that you played a sport you were not supposed to.”*	Categorizing the child.	*“You are irresponsible, I always have to check up on you.”*

This table was adapted from the work of Koestner, Ryan and Bernieri [29], and Faber and Mazlish [43]

## Conclusions

In summary, recent research has shown it can be difficult for children with hemophilia and their parents to implement the recommendations of the healthcare team into their daily life. Challenges are even greater during the transition from parent-care to self-care. Parents’ systematic use of various communication strategies to help promote autonomy and appropriate self-care in young patients can be beneficial. Such strategies can help parents promote autonomy in their child while maintaining necessary boundaries. As illustrated in Table 1, the self-determination approach provides tools to help promote self-motivation in children with hemophilia during the transition towards self-care. We believe that understanding and using these communication principles can help caregivers better address the motivation challenges in self-care faced by children and adolescents with hemophilia. Future research initiatives should address the development of standardized caregiver training following these principles and offer appropriate strategy to evaluate it.

## References

[1] McLaughlin J, Witkop M, Lambing A, Anderson T, Munn J, Tortella B (2014). Better adherence to prescribed treatment regimen is related to less chronic pain among adolescents and young adults with moderate or severe haemophilia. Haemophilia.

[2] García-Dasí M, Aznar J, Jiménez-Yuste V, Altisent C, Bonanad S, Mingot E (2015). Adherence to prophylaxis and quality of life in children and adolescents with severe haemophilia A. Haemophilia.

[3] Taddeo D, Egedy M, Frappier J-Y (2008). Adherence to treatment in adolescents. Paediatr Child Health.

[4] Geraghty S, Dunkley T, Harrington C, Lindvall K, Maahs J, Sek J (2006). Practice patterns in haemophilia A therapy -- global progress towards optimal care. Haemophilia.

[5] Schrijvers L, Uitslager N, Schuurmans M, Fischer K (2013). Barriers and motivators of adherence to prophylactic treatment in haemophilia: a systematic review. Haemophilia.

[6] Nazzaro A-M, Owens S, Hoots WK, Larson KL (2006). Knowledge, attitudes, and behaviors of youths in the US hemophilia population: results of a national survey. Am J Public Health.

[7] Hacker M, Geraghty S, Manco‐Johnson M (2001). Barriers to compliance with prophylaxis therapy in haemophilia. Haemophilia.

[8] De Moerloose P, Urbancik W, Van Den Berg HM, Richards M (2008). A survey of adherence to haemophilia therapy in six European countries: results and recommendations. Haemophilia.

[9] Kahana S, Drotar D, Frazier T (2008). Meta-analysis of psychological interventions to promote adherence to treatment in pediatric chronic health conditions. J Pediatr Psychol.

[10] Young G (2012). From boy to man: recommendations for the transition process in haemophilia. Haemophilia.

[11] Chan DK, Lonsdale C, Ho PY, Yung PS, Chan KM (2009). Patient motivation and adherence to postsurgery rehabilitation exercise recommendations: the influence of physiotherapists’ autonomy-supportive behaviors. Arch Phys Med Rehabil.

[12] Srivastava A, Brewer A, Mauser-Bunschoten E, Key N, Kitchen S, Llinas A (2013). Guidelines for the management of hemophilia. Haemophilia.

[13] Ross C, Goldenberg NA, Hund D, Manco-Johnson MJ (2009). Athletic participation in severe hemophilia: bleeding and joint outcomes in children on prophylaxis. Pediatrics.

[14] Fromme A, Dreeskamp K, Pollmann H, Thorwesten L, Mooren F, Völker K (2007). Participation in sports and physical activity of haemophilia patients. Haemophilia.

[15] Duncan N, Shapiro A, Ye X, Epstein J, Luo M (2012). Treatment patterns, health-related quality of life and adherence to prophylaxis among haemophilia A patients in the United States. Haemophilia.

[16] Morris AD, Boyle DI, McMahon AD, Greene SA, MacDonald TM, Newton RW (1997). Adherence to insulin treatment, glycaemic control, and ketoacidosis in insulin-dependent diabetes mellitus. Lancet.

[17] Jonasson G, Carlsen K, Sodal A, Jonasson C, Mowinckel P (1999). Patient compliance in a clinical trial with inhaled budesonide in children with mild asthma. Eur Respir J.

[18] Austin S, Senécal C, Guay F, Nouwen A (2011). Effects of gender, age, and diabetes duration on dietary self-care in adolescents with type 1 diabetes: a self-determination theory perspective. J Health Psychol.

[19] Austin S, Guay F, Senécal C, Fernet C, Nouwen A (2013). Longitudinal testing of a dietary self-care motivational model in adolescents with diabetes. J Psychosom Res.

[20] Lindvall K, Colstrup L, Wollter IM, Klemenz G, Loogna K, Grönhaug S (2006). Compliance with treatment and understanding of own disease in patients with severe and moderate haemophilia. Haemophilia.

[21] Markova I, Macdonald K, Forbes C (1980). Impact of haemophilia on child-rearing practices and parental co-operation. J Child Psychol Psychiatry.

[22] Holmbeck GN, Johnson SZ, Wills KE, McKernon W, Rose B, Erklin S (2002). Observed and perceived parental overprotection in relation to psychosocial adjustment in preadolescents with a physical disability: the mediational role of behavioral autonomy. J Consult Clin Psychol.

[23] Mak AS (1994). Parental neglect and overprotection as risk factors in delinquency. Aust J Psychol.

[24] Cimarolli VR, Reinhardt JP, Horowitz A (2006). Perceived overprotection: support gone bad?. J Gerontol B Psychol Sci Soc Sci.

[25] Janssens KA, Oldehinkel AJ, Rosmalen JG (2009). Parental overprotection predicts the development of functional somatic symptoms in young adolescents. J Pediatr.

[26] Eiser C. The psychology of childhood illness. Springer Science & Business Media. New-York; 2012.

[27] Dutreil S, Rice J, Merritt D, Kuebler E (2011). Parents Empowering Parents (PEP) Program: understanding its impact on the bleeding disorders community. Haemophilia.

[28] Deci EL, Ryan RM. Handbook of self-determination research. Rochester: University Rochester Press; 2002.

[29] Koestner R, Ryan RM, Bernieri F, Holt K (1984). Setting limits on children’s behavior: the differential effects of controlling vs. informational styles on intrinsic motivation and creativity. J Pers.

[30] Grolnick WS, Deci EL, Ryan RM (1997). Internalization within the family: the self-determination theory perspective.

[31] Ryan RM, Deci EL (2000). Self-determination theory and the facilitation of intrinsic motivation, social development, and well-being. Am Psychol.

[32] Ryan RM, Deci EL (2000). Intrinsic and extrinsic motivations: classic definitions and new directions. Contemp Educ Psychol.

[33] Hagger MS, Chatzisarantis NL (2009). Integrating the theory of planned behaviour and self‐determination theory in health behaviour: a meta‐analysis. Br J Health Psychol.

[34] Rutten C, Boen F, Seghers J (2013). The relation between environmental factors and pedometer-determined physical activity in children: the mediating role of autonomous motivation. Pediatr Exerc Sci.

[35] Ryan RM, Deci EL, Grolnick WS, La Guardia JG. The significance of autonomy and autonomy support in psychological development and psychopathology. 2006.

[36] Joussemet M, Landry R, Koestner R (2008). A self-determination theory perspective on parenting. Can Psychol.

[37] Grolnick WS, Ryan RM (1989). Parent styles associated with children’s self-regulation and competence in school. J Educ Psychol.

[38] Joussemet M, Koestner R, Lekes N, Landry R (2005). A longitudinal study of the relationship of maternal autonomy support to children’s adjustment and achievement in school. J Pers.

[39] Grolnick WS, Gurland ST, DeCourcey W, Jacob K (2002). Antecedents and consequences of mothers’ autonomy support: an experimental investigation. Dev Psychol.

[40] Grolnick WS, Deci EL, Ryan RM, Grusec JE, Kuczynski L (1997). Internalization within the family: the self-determination theory perspective. Parenting and children’s internalization of values: a handbook of contemporary theory.

[41] Chatzisarantis NL, Hagger MS (2009). Effects of an intervention based on self-determination theory on self-reported leisure-time physical activity participation. Psychol Health.

[42] Joussemet M, Mageau GA, Koestner R (2014). Promoting optimal parenting and children’s mental health: a preliminary evaluation of the How-to parenting program. J Child Fam Stud.

[43] Faber A, Mazlish E. How to talk so kids will listen & listen so kids will talk. 2012: Simon and Schuster, New-York.

[44] Reeve J, Jang H (2006). What teachers say and do to support students’ autonomy during a learning activity. J Educ Psychol.

[45] Wu YP, Rausch J, Rohan JM, Hood KK, Pendley JS, Delamater A (2014). Autonomy support and responsibility-sharing predict blood glucose monitoring frequency among youth with diabetes. Health Psychol.

[46] Williams GC, Deci EL (2001). Activating patients for smoking cessation through physician autonomy support. Med Care.

[47] Williams GG, Gagné M, Ryan RM, Deci EL (2002). Facilitating autonomous motivation for smoking cessation. Health Psychol.

